# Adoptive Transfer of Mammaglobin-A Epitope Specific CD8 T Cells Combined with a Single Low Dose of Total Body Irradiation Eradicates Breast Tumors

**DOI:** 10.1371/journal.pone.0041240

**Published:** 2012-07-20

**Authors:** Nadine M. Lerret, Magdalena Rogozinska, Andrés Jaramillo, Amanda L. Marzo

**Affiliations:** 1 Rush University Medical Center, Department of Immunology and Microbiology, Chicago, Illinois, United States of America; 2 Rush University Medical Center, Department of Pathology, Chicago, Illinois, United States of America; 3 Histocompatibility Laboratory, Gift of Hope Organ & Tissue Donor Network, Itasca, Illinois, United States of America; University of Nebraska Medical Center, United States of America

## Abstract

Adoptive T cell therapy has proven to be beneficial in a number of tumor systems by targeting the relevant tumor antigen. The tumor antigen targeted in our model is Mammaglobin-A, expressed by approximately 80% of human breast tumors. Here we evaluated the use of adoptively transferred Mammaglobin-A specific CD8 T cells in combination with low dose irradiation to induce breast tumor rejection and prevent relapse. We show Mammaglobin-A specific CD8 T cells generated by DNA vaccination with all epitopes (Mammaglobin-A2.1, A2.2, A2.4 and A2.6) and full-length DNA *in vivo* resulted in heterogeneous T cell populations consisting of both effector and central memory CD8 T cell subsets. Adoptive transfer of spleen cells from all Mammaglobin-A2 immunized mice into tumor-bearing SCID/beige mice induced tumor regression but this anti-tumor response was not sustained long-term. Additionally, we demonstrate that only the adoptive transfer of Mammaglobin-A2 specific CD8 T cells in combination with a single low dose of irradiation prevents tumors from recurring. More importantly we show that this single dose of irradiation results in the down regulation of the macrophage scavenger receptor 1 on dendritic cells within the tumor and reduces lipid uptake by tumor resident dendritic cells potentially enabling the dendritic cells to present tumor antigen more efficiently and aid in tumor clearance. These data reveal the potential for adoptive transfer combined with a single low dose of total body irradiation as a suitable therapy for the treatment of established breast tumors and the prevention of tumor recurrence.

## Introduction

Two major goals in tumor vaccination are to induce regression of established tumors and to generate long-lasting tumor-specific immunity capable of protecting the host from relapse. The ability to confer long-term protection in cancer has been limited largely by the inability to generate effective memory CD8 T cell responses. Memory CD8 T cells are endowed with unique properties that permit more vigorous and specific responses upon re-challenge to protect against tumor challenge. Memory CD8 T cells are long-lived cells with heightened capacity to respond to subsequent insults that have been divided into two sub-populations, central memory T cells (T_CM_) and effector memory T cells (T_EM_), based on their expression of CCR7 and CD62L [1]. Ideally DNA vaccination would induce a robust population of both memory T cell subsets with the T_EM_ acting as the initial defenders against tumor challenge and T_CM_ replenishing the pool of T_EM_. Nonetheless, DNA vaccination in the cancer setting has often failed due to the lack of T cell antigens expressed by cancer cells. More recently, Mammaglobin-A (Mam-A) has been found to be a clinically relevant breast cancer-associated antigen that is over-expressed in both human breast cancer cell lines and primary human breast tumors [2]. Mam-A expression is similar on well-differentiated, moderately differentiated, and poorly differentiated primary breast tumors [2], [3]. Furthermore, this protein is frequently produced by metastatic breast cancer cells [2]. Previously, CD8 T cells generated by vaccination with full-length Mam-A DNA were able to specifically induce the regression of Mam-A^+^ breast cancer tumors *in vivo* [3], [4] but their ability to provide long-term tumor specific immunity was not determined. These studies also demonstrated that CD8 T cells from HLA-A2^+^ breast cancer patients and vaccinated HLA-A2^+^ transgenic mice recognized a similar epitope profile derived from the Mam-A protein [3], [4]. In this regard, HLA-A2^+^ transgenic mice have been previously shown to recognize similar epitopes from influenza virus, hepatitis C virus, cytomegalovirus, and leukemia-associated antigens as HLA-A2^+^ humans [5], [6], [7], [8]. Four epitopes derived from Mam-A (Mam-A2.1, A2.2, A2.4, and A2.6) have been identified that are recognized by vaccinated HLA-A2^+^ transgenic mice [3], [4]. However, whether or not these epitope-specific CD8 T cells could lead to *in vivo* tumor regression and prevent tumor recurrence was not determined. Thus our objective was to determine if vaccination with any of the Mam-A epitope cDNA constructs would result in a more effective and prolonged CD8 CTL response against breast cancer cells and if not, could a single low dose of irradiation be used in combination with adoptive therapy to induce an effective and sustainable anti-tumor immune response.

Therefore we used adoptive cell transfer of Mam-A specific T cells as an immunotherapeutic strategy aimed at over-coming the poor natural T-cell response to tumors [9], [10] and show that even the most functional Mam-A2 specific CTL's, although initially able to induce tumor regression, were unable to sustain long term tumor specific immunity. Consequently, we determined if a single low dose of irradiation (as opposed to higher doses or multiple low doses of irradiation which have numerous undesirable side effects) would augment the anti-tumor response of the adoptively transferred Mam-A tumor-specific T cells. This line of therapy was selected because current cancer therapy often incorporates lymphodepleting regimes as a way to augment adoptive transfer of tumor-specific T cells by removing the host's suppressor cells such as T-regulatory cells (T-regs) and myeloid-derived suppressor cells (MDSCs) [11]. Lymphodepletion also removes cytokine sinks and induces the release of Toll like receptor agonists that activate the innate immune system [11], [12], [13]. Thus, in the present study we treated tumor-bearing SCID-beige mice with a single low dose of total body irradiation (TBI) immediately prior to the transfer of Mam-A-epitope specific splenocytes. We show that by combining Mam-A2 tumor-specific CD8 T cells with a single low dose of TBI we were able to induce successful breast tumor regression, and, unlike previous modalities tested, prevent tumor re-growth. Our data suggest an additional mechanism by which TBI enhances the anti-tumor response is by altering the biology of tumor derived dendritic cells (DCs). We show that TBI not only acts to inhibit tumor growth directly but also results in the down regulation of the macrophage scavenger receptor and reduces lipid uptake by tumor resident DC, both events that can increase the functional capacity of DCs.

## Results

### DNA vaccination with all Mam-A epitopes generates a heterogeneous population of CD8 T cells consisting of both central and effector memory CD8 T cells

**Figure 1:**
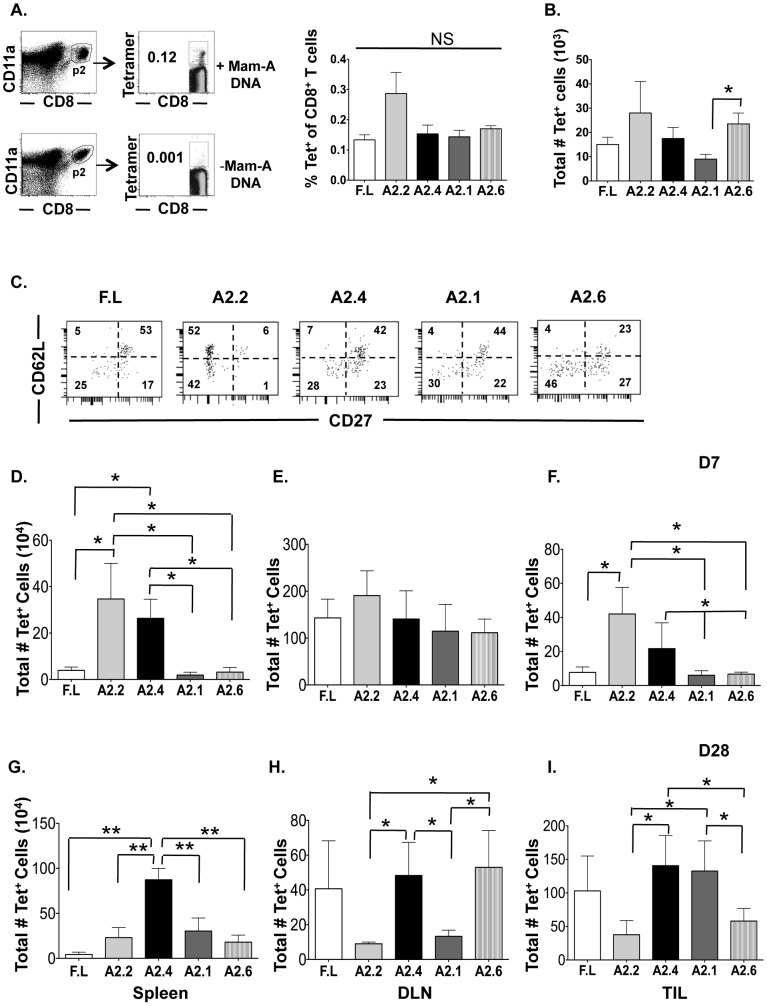
DNA vaccination with all Mam-A epitopes generates a heterogeneous population of Mam-A2 specific CD8 T cells that are maintained at various frequencies in vivo. (A) HLA-A2^+^ transgenic mice vaccinated four times separated by 2-week intervals with Mam-A encoded cDNA. 5 days after the last vaccination, animals were sacrificed and spleen cells were harvested. As a negative control, spleen cells were also harvested from unvaccinated HLA-A2^+^ transgenic mice. Lymphocytes were stained and CD8^+^CD11a^hi^ T cells were gated on first and then the tetramer +ve CD8 T cells were gated and this gate was used to determine the percent of Mam-A tetramer positive of CD8 T cells and (B) the total number of tetramer positive CD8 T cells. A representative gating scheme for all groups is shown. Only the Mam-A tetramer positive cells were collected via flow cytometry for further experiments due to the low frequency of these cells present 5 days after the last vaccination (C) The expression of CD27 and CD62L on splenic Mam-A tetramer positive CD8 T cells was determined five days after the last vaccination with cDNA encoding for either full-length or epitope specific Mam-A. 1×10^7^ spleen cells from HLA-A2^+^ transgenic mice vaccinated with cDNA encoding for either full-length or epitope-specific Mam-A were injected i.p into tumor-bearing SCID-beige mice. (D–F) 7 and (G–I) 28 days after transfer, animals were sacrificed and the total number of tetramer positive of CD8 T cells was determined in the (D, G) spleen, (E, H) draining lymph node and (F, I) tumor from the CD8^+^CD11a^hi^ T cell population. Each bar represents the mean (± SEM) of Mam-A-tetramer+ cells and is representative of the data collected from 8–9 mice. ^*^ P<0.05 and ** P<0.01.

To study the effect of Mam-A DNA vaccination on the generation of long-lived effector CD8 T cells we enumerated and phenotyped the Mam-A specific CD8 T cells generated after vaccination with full-length or Mam-A epitope encoded DNA. HLA-A2^+^ transgenic mice were vaccinated four times with Mam-A DNA (full-length or epitopes). Five days after the last vaccination, the percent of tetramer positive of Mam-A specific CD8^+^CD11a^hi^ T cells were found to be similar amongst the different Mam-A epitopes and full-length DNA vaccinations (Fig. 1a). Furthermore, when total numbers of Mam-A^+^ tetramer^+^ CD8^+^CD11a^hi^ T cells were analyzed, Mam-A2.2, Mam-A2.4, Mam-A2.6 and full-length DNA vaccination induced similar numbers of total Mam-A specific CD8 T cells while Mam-A2.1 DNA induced a slightly smaller number of these cells (Fig. 1b). We then evaluated the phenotype of Mam-A tetramer specific CD8^+^CD11a^hi^ T cells generated five days after the last vaccination with full-length, Mam-A2.1, Mam-A2.2, Mam-A2.4 or Mam-A2.6 epitope encoded DNA (Fig. 1c). CD62L is often used to delineate T_CM_ from T_EM_ CD8 T cells and its expression is necessary for lymphocytes to enter lymphoid tissues. Expression of CD62L is lower on T_EM_, which is consistent with their ability to home to peripheral non-lymphoid tissues [1], [14], [15], [16]. CD27 is a cell surface marker belonging to the tumor-necrosis factor receptor (TNFR) superfamily that is constitutively expressed on naïve CD8 T cells and expression is higher on CD8 T cells in secondary lymphoid tissues after T cell activation and remains high on T_CM_ CD8 T cells and is down-regulated on T cells when they enter non-lymphoid tissues [17], [18], [19]. CD27 can also be used to further distinguish T_EM_ CD8 T cells into two subsets, CD62L^lo^/CD27^lo^ and CD26L^lo^/CD27^hi^ [19]. Vaccination with full-length Mam-A, Mam- A2.4 and Mam-A2.1 DNA induced similar numbers of Mam-A specific CD27^+^/CD62L^+^ CD8 T_CM_ (53,42 and 44%, respectively), indicating adequate generation of CD8 T_CM_ (Fig. 1c). However, vaccination with Mam-A2.2 and Mam-A2.6 DNA lead to the generation of fewer T_CM_ than the other epitopes (6 and 23%, respectively). When examining the two subsets of T_EM_ defined by CD27 expression, vaccination with full-length Mam-A, Mam-A2.4 and Mam-A2.1 induced similar numbers of CD62L^lo^/CD27^lo^ Mam-A specific CD8 T cells (25,28 and 30%, respectively) whereas vaccination with Mam-A2.2 and Mam-A2.6 induced slightly more CD62L^lo^/CD27^lo^ Mam-A specific CD8 T cells (42 and 46% respectively). Interestingly, vaccination with Mam-A2.2 DNA failed to induce the CD62L^lo^/CD27^hi^ population. In contrast, vaccination with the full-length Mam-A, Mam-A2.4, Mam-A 2.1 and Mam-A2.6 induced Mam-A specific CD8 T cells with a CD62L^lo^/CD27^hi^ phenotype (17, 23, 22 and 27%, respectively). The cell surface marker CD122, defined as the β-chain of IL-2R and IL-15R, confers responsiveness to IL-15, a cytokine crucial for the maintenance of memory CD8 T cells induced through homeostatic proliferation [20]. Analysis of CD122 expression revealed that DNA vaccination with Mam-A2.4 was capable of inducing a slightly higher percentage of CD122^+^CD8^+^ T cells (24%) compared to full-length Mam-A, Mam-A2.2, Mam-A2.1 and Mam-A2.6 (16,13, 9 and14% respectively, Fig.S1). Additionally, CD127 (IL-7Rα) is important for development and survival of memory CD8 T cells and is highly expressed on a subset of effector CD8 T cells known as memory precursor effector cells (MPECs) that eventually become long-lived T_EM_ [21], [22], [23] [24]. Mam-A2.4 DNA vaccination induced a slightly larger percentage of antigen-specific CD127^+^ CD8 T cells when compared to Mam-A epitopes 2.1,2.2 and 2.6 or full-length vaccination (Fig.S1). These results indicate that in our model, while vaccination with all epitopes and full-length Mam-A DNA can induce effector CD8 T cells, Mam-A2.2 and Mam-A2.4 specific CD8 T cells have an increased capacity to be maintained and respond more effectively in subsequent tumor challenges as a result of their increased levels of CD122.

We subsequently evaluated the functional capacity of Mam-A specific CD8 T cells by measuring their ability to produce IFN-γ in response to a single Mam-A epitope (Mam-A2.1 A2.2, A2.4, A2.6 or a combination of all epitopes (full-length Mam-A equivalent). Vaccination with all Mam-A epitopes induced equal amounts of IFN-γ production (Fig.S1). In contrast, vaccination with full-length Mam-A was unable to induce IFN-γ-producing cytotoxic CD8 T cells. TNF-α production was also analyzed after vaccination with full-length and Mam-A epitope DNA, there were no significant difference in TNF-α production when compared to the isotype control in any of the Mam-A tetramer^+^ T cells (data not shown). As most tumor-associated antigens are derived from a mutated form of self and since T-regs play a key role in maintaining self-tolerance, we analyzed whether or not vaccination with either full-length or epitope encoded Mam-A DNA induced higher numbers of T-regs (CD4^+^CD25^+^FoxP3^+^). While relatively similar numbers of T-regs were produced following the majority of immunization regimes, full-length Mam-A induced a slightly smaller number of T-regs (Fig.S1). Taken together, these data indicate that DNA vaccination with all epitopes and full-length Mam-A DNA can induce a heterogeneous population of CD8 T cells (Fig. 1 and Fig.S1).

We next enumerated the total number of Mam-A-tetramer^+^ of CD8^+^CD11a^hi^ T cells from the spleen, DLN and tumor of SCID-beige tumor bearing mice 7 and 28 days after the transfer of splenocytes from Mam-A DNA vaccinated HLA-A2^+^ transgenic mice. Mam-A2.2 and A2.4 specific CD8 T cells were maintained at a higher frequency in the spleen 7 days post transfer than either full length or the other epitopes (Fig. 1d). In contrast, in all vaccination groups, a similar number of Mam-A tetramer+ CD8 T cells were present in the DLN 7 days post transfer (Fig. 1e). When analyzing the tumor infiltrating Mam-A specific CD8 T cells 7 days post transfer, mice that had received splenocytes from Mam-A2.2 and Mam-A2.4 vaccinated mice were found to have a larger number of Mam-A specific tetramer+ CD8 T cells compared to Mam-A2.1, Mam-A2.6 and full-length vaccinated mice (Fig. 1f). By day 28 post transfer, the total number of Mam-A2.4 specific CD8 T cells found within the spleen was significantly higher than the other Mam-A epitopes or full-length Mam-A specific CD8 T cells (Fig. 1g). However, by day 28 in the DLN, Mam-A2.4 and Mam-A2.6 specific CD8 T cells were present at significantly higher numbers than the other Mam-A epitopes (Fig. 1h). Importantly, there were three times as many Mam-A2.4 specific CD8 T cells in the tumor at day 28 than Mam-A2.2 or Mam-A2.6 specific CD8 T cells (Fig. 1i).

**Figure 2:**
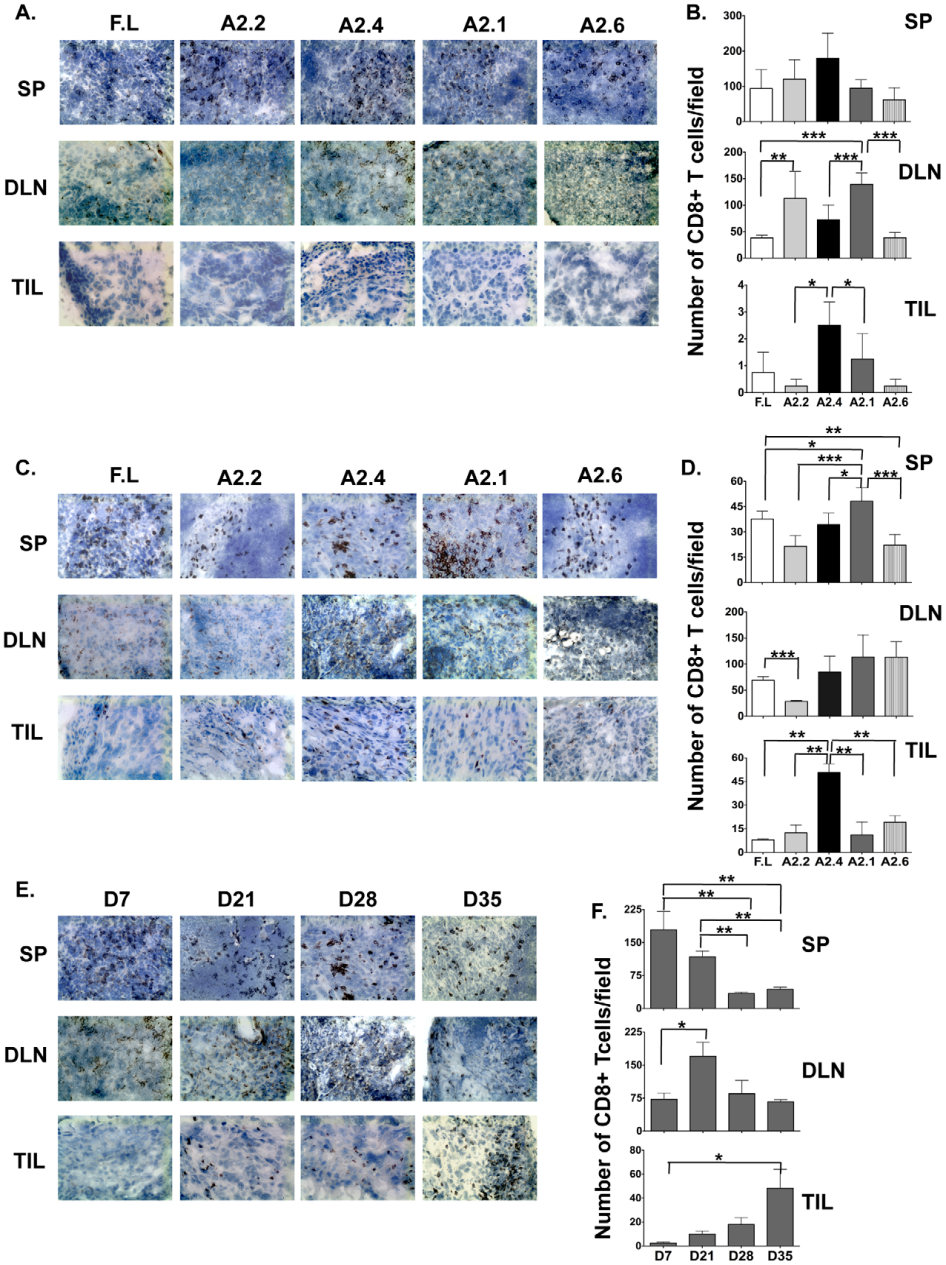
CD8^+^ T cells from Mam-A2.4 DNA vaccinated HLA-A2^+^ transgenic mice traffic into breast tumors at a higher frequency. Spleen (SP), draining lymph node (DLN) and tumor (TIL) sections from SCID-beige mice receiving splenocytes from HLA-A2^+^ transgenic mice vaccinated with cDNA encoding either full-length or Mam-A epitopes were analyzed by means of immunohistochemistry for the presence of CD8 T cells (A) 7, and (C) 28 days post transfer (magnification of 20×). Morphometric analysis was also performed (B) 7 and (D) 28 days post transfer. Results are expressed as the mean (±SEM) of 3 different experiments. (E–F) Spleen (SP), draining lymph node (DLN) and tumor (TIL) sections from SCID-beige mice receiving splenocytes from HLA-A2^+^ transgenic mice vaccinated with cDNA encoding Mam-A2.4 were analyzed for the presence of CD8 T cells on days 7, 21, 28 and 35 post spleen cell transfer. Data are representative of at least 2 experiments with at least 3 mice per group/experiment. ^*^ P<0.05, ** P<0.01 and *** P<0.0001.

Immunohistochemical analysis revealed that CD8 T cells from animals vaccinated with DNA encoding for all Mam-A epitopes as well as full-length Mam-A infiltrated the spleens and DLN but only CD8 T cells from Mam-A2.4 infiltrated the tumor on day 7 (Fig. 2a–2b). By day 28, infiltration of CD8 T cells was observed in the tumor from mice receiving spleen cells from all vaccination groups (Fig. 2c–2d). However, mice receiving spleen cells from Mam-A2.4 DNA vaccinated mice were observed to have the highest number of tumor infiltrating CD8 T cells on day 28 and these cells were still present at day 35 (Fig. 2c–2f). Morphometric analysis confirmed the increased presence of CD8 T cells in mice receiving spleen cells from Mam-A2.4 DNA vaccinated mice (Fig. 2f).

### Mam-A2 specific CD8 T cells that accumulate in the tumor earlier and maintained longer are predominantly CD27lo/CD62Llo/CD127hi

**Figure 3:**
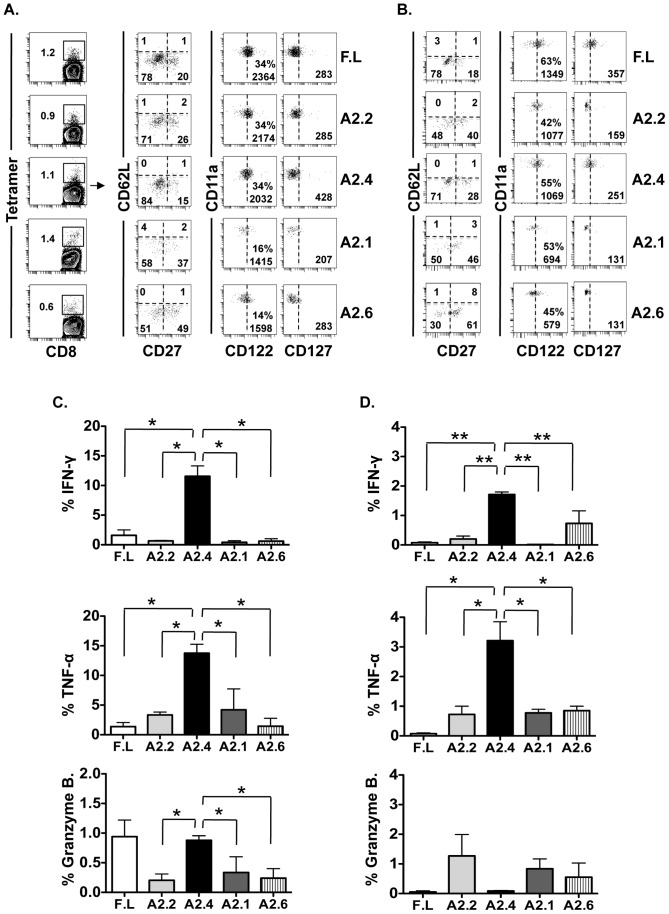
CD8 T cells from Mam-A2.4 DNA vaccinated HLA-A2^+^ transgenic mice are significantly more cytotoxic. Tumor-bearing SCID-beige mice received spleen cells (1×10^7^ i.p) from HLA-A2^+^ transgenic mice that were vaccinated with cDNA encoding for either full-length or epitope-specific Mam-A. (A) Animals were sacrificed 7 or (B) 28 days later and Mam-A tetramer-specific CD11a^hi^CD8 T cells from the spleens were analyzed via flow cytometry for CD122, CD127, CD27, and CD62L expression. Data are representative of at least 3 experiments with 3–4 mice per group. Mam-A specific T cells were analyzed for IFN-γ, TNF-α and granzyme B production after stimulation with 40 µg/ml of the corresponding Mam-A peptides (C) 7 and (D) 28 days post adoptive transfer of Mam-A specific splenocytes. The percentage of antigen specific CD11a^hi^ CD8^+^ T cells that secrete IFN-γ, TNF-α or Granzyme B in response to peptide stimulation is shown. ^*^ P<0.05 and ** P<0.01. Data are representative of 2 experiments with at least 3–4 mice per group.

We next analyzed the phenotype and function of the Mam-A tetramer^+^ CD8^+^CD11a^hi^ T cells from the spleens of SCID-beige tumor bearing mice. By day 7 post transfer there were no Mam-A specific CD8 T_CM_ (CD27^hi^/CD62L^hi^) present in the spleens of mice that received either full-length Mam-A or any of the Mam-A epitopes (Fig. 3a). Although the percentage of Mam-A specific CD8 T_EM_ defined as CD62L^lo^ were the same amongst the different experimental groups 7 days post transfer, analysis of the T_EM_ subsets (defined by the expression of CD27 and CD62L) revealed that the majority of Mam-A specific CD8 T cells from mice that received splenocytes from full-length, Mam-A2.2 and Mam-A2.4 vaccinated mice 7 days prior were of the CD27^lo^/CD62L^lo^ T_EM_ phenotype (78, 71 and 84%, respectively). On the other hand, mice that had received either Mam-A2.1 or Mam-A2.6 had fewer CD27^lo^/CD62L^lo^ CD8 T_EM_ cells (58 and 51%, respectively) with a proportionally larger population of CD27^hi^/CD62L^lo^ CD8 T_EM_ cells. Analysis of CD122 expression revealed similar MFI levels amongst CD8 T cells from mice vaccinated with full-length Mam-A, Mam-A2.2 and Mam-A2.4 with slightly lower MFIs for CD8 T cells from mice vaccinated with Mam-A2.1 and Mam-A2.6. In contrast, CD127 expression was higher in the animals that received Mam-A2.4 compared to full-length, Mam-A2.2, Mam-A2.1 and Mam-A2.6 (428, 283, 285, 207 and 200, respectively), consistent with their ability to persist long-term. By day 28 the CD62L profiles were similar to that at day 7 (Fig. 3b). However, by this time-point more of the CD62L^lo^ Mam-A epitope specific CD8 T cells expressed CD27 compared to day 7 (Fig. 3b). While CD122 expression on CD8 T cells was similar amongst the groups, CD8 T cells from mice vaccinated with full-length and Mam-A2.4 specific CD8 T cells expressed slightly more CD127 than CD8 T cells from mice vaccinated with the other Mam-A epitopes (MFI of 357, 251, 159, 111 and 131, respectively).

### CD27^lo^/CD62L^lo^/CD127^hi^ Mam-A2 specific CD8 T cells (Mam-A2.4) secrete more IFN-γ, TNF-α and express more Granzyme B post transfer than other Mam-A epitope specific CD8 T cells

The capacity of these Mam-A specific CD8 T cells to secrete IFN-γ, TNF-α and express Granzyme B on days 7 and 28 post transfer was analyzed (Fig. 3c–3d). Even though CD8 T cells derived from mice vaccinated with either full-length or Mam-A epitope DNA secreted similar amounts of both IFN-γ and TNF-α before transfer (Fig S1 and data not shown) 7 days after transfer, Mam-A2.4 specific CD8 T cells secreted at least 5 times more IFN-γ and at least twice as much TNF-α when compared to CD8 T cells specific for the other Mam-A epitopes or full-length Mam-A (Fig. 3c). In addition, Mam-A2.4 specific CD8 T cells also expressed at least 3 times more Granzyme B than CD8 T cells specific for the other epitopes (Fig. 3c). While similar numbers of Mam-A2.2 CD8 T cells were seen at day 7 post transfer when compared to Mam-A2.4 CD8 T cells (Fig. 1d) these Mam-A2.2 specific CD8 T cells secreted less IFN-γ, TNF-α and expressed less Granzyme B compared to Mam-A2.4 CD8 T cells (Fig. 3c). By day 28 after transfer, the Mam-A2.4 specific CD8 T cells continued to secrete significantly more IFN-γ and TNF-α compared to CD8 T cells specific for either full-length or the other Mam-A epitopes (Fig. 3d).

### CD8 T cells from mice vaccinated with Mam-A2.4 DNA induce long-term tumor regression and survival *in vivo* when compared to CD8 T cells from mice vaccinated with full-length Mam-A DNA or other Mam-A epitope DNA but are unable to prevent recurrence of tumor

**Figure 4:**
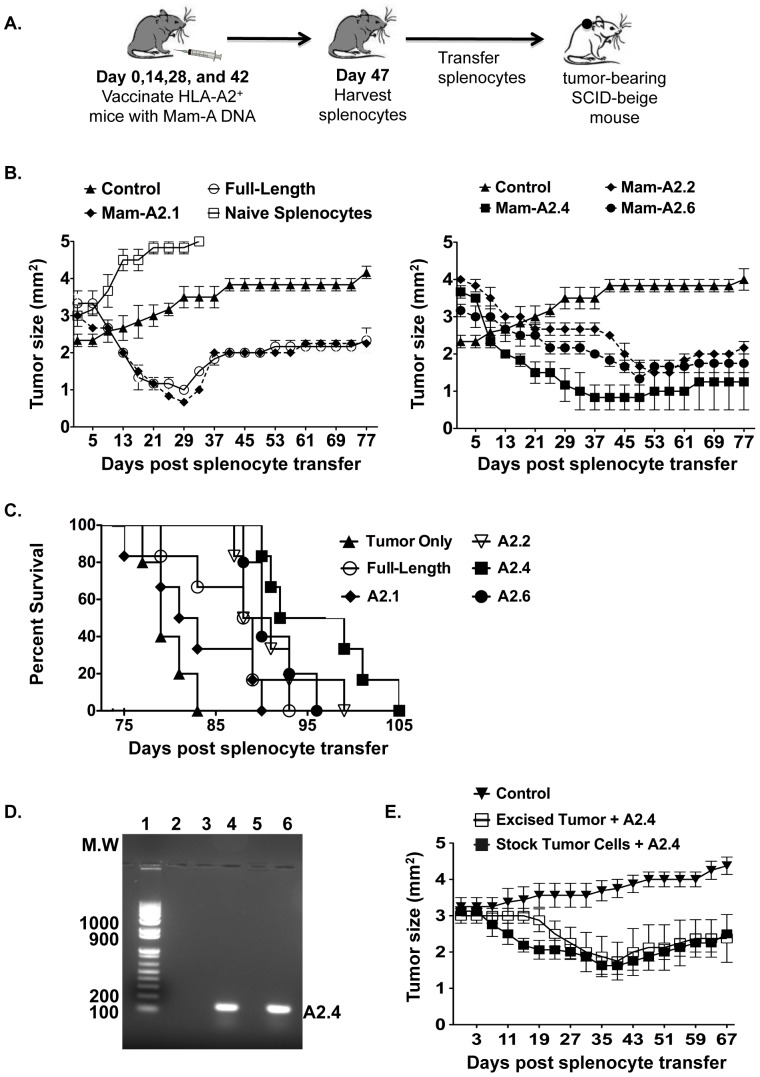
DNA vaccination with Mam-A2.4 induces tumor regression and prolongs survival *in vivo*. (A) HLA-A2^+^ transgenic mice were vaccinated i.m a total of 4 times, separated by 2 week intervals, with 100 µg Mam-A full-length or epitope specific cDNA. Five days after the last vaccination 1×10^7^ Mam-A full-length or epitope specific spleen cells were harvested and injected i.p into SCID-beige mice bearing tumors that were approximately 4 mm^2^. (B) Tumor regression and (C) surival was monitored in mice that received either splenocytes from non-vaccinated mice, tumor alone, full-length Mam-A, Mam-A2.1 or Mam-A2.2, Mam-A2.4 Mam-A2.6 specific spleen cells. Results representative of 4 independent experiments with n = 4 mice/group (D) Mam-A expression was determined by PCR from the AU-565 tumor cell line either before transplant or 35 days after transplant following adoptive transfer of Mam-A2.4 splenic T cells. Lane 1 = molecular weight marker, lane 2 = blank, lane 3 = RNA from AU-565 tumor prior to transplant without reverse transcriptase (negative control), lane 4 = RNA from AU-565 tumor prior to transplant, lane 5 = RNA from AU-565 tumor 35 days after transplant without reverse transcriptase (negative control), lane 6 = RNA from AU-565 tumor 35 days post transplant. (E) Single cell suspensions were made from tumors excised from SCID-beige mice that received Mam-A2.4 specific splenocytes 35 days prior. Tumor cell suspensions were cultured for 7–10 days and then injected into SCID-beige mice. Once tumors were established (4 mm^2^), mice were reconstituted with splenocytes from Mam-A2.4 vaccinated HLA-A2^+^ transgenic mice and tumor regression monitored. Control mice received the original tumor cell line. Data are representative of 3 experiments.

While we have shown that vaccination of HLA-A2^+^ transgenic mice with Mam-A2.4 encoded DNA produces splenocytes that have the phenotype and functional attributes required for a successful long-lived antitumor response (Fig. 1 and Fig S1), their ability to cause tumor regression/rejection *in vivo* thus far is unknown. Therefore, we next transferred splenocytes from Mam-A vaccinated HLA-A2^+^ transgenic mice into tumor-bearing SCID-beige mice and monitored tumor regression for 77 days (Fig. 4a–b). While all four of the Mam-A epitopes and full-length Mam-A initially induced successful tumor regression, tumors in mice receiving splenocytes from Mam-A2.2, Mam-A2.4 and Mam-A2.6 DNA vaccinated mice were capable of inducing longer-term tumor regression with Mam-A2.4 showing the greatest decrease in tumor volume, prolonged tumor remission and survival (Fig. 4b–c and Fig S2). Because all the Mam-A splenocytes induced some degree of tumor regression we explored the possibility that allorecognition was contributing to the tumor rejection. Therefore, we transferred naïve splenocytes from non-immunized HLA-A2 transgenic mice into tumor-bearing SCID-beige mice and monitored tumor regression (Fig. 4a). Unlike the transfer of splenocytes from Mam-A vaccinated HLA-A2 transgenic mice, the transfer of splenocytes from naïve HLA-A2^+^ transgenic mice failed to induce tumor regression. Nonetheless, even if allorecognition is playing a role in the rejection process, tumors in mice receiving splenocytes from Mam-A vaccinated mice eventually grew back indicating that although CD8 T cells specific for Mam-A2 initially induce tumor regression *in vivo*, this vaccination regime alone isn't sufficient to prevent tumor re-growth.

The observation of tumor re-growth may indicate possible escape by the tumor cells through loss of tumor antigen. To determine if tumors retained their expression of Mam-A after they grew back, RNA was extracted from tumors isolated on day 37 from mice that received Mam-A2.4 specific splenocytes and the detection of Mam-A antigen was determined by PCR. Figure 4d demonstrates that tumors that grew back still expressed the Mam-A antigen. Additionally, we show that tumor cell lines derived from animals where tumors had previously grown back were equally capable of regressing as compared to the original Mam-A^+^ tumor cell line (Fig. 4e). Together these results indicate that loss of tumor antigen is not responsible for tumor outgrowth. Moreover, with the transfer of whole splenocytes antigen-specific CD8 T cells as well as regulatory T cells (CD4^+^CD25^+^ T cells) are introduced into the tumor-bearing host. However, when we depleted CD4 T cells prior to adoptive transfer of Mam-A2.4 specific splenocytes, the recipient SCID-beige mice did not show a further decrease in tumor burden (Fig S3), indicating that regulatory CD4 T cells are not responsible for tumor escape in our model.

### CD4 T cells are not required for effector Mam-A2.4 specific CD8 T cells to enter the tumor site and cause tumor regression

**Figure 5:**
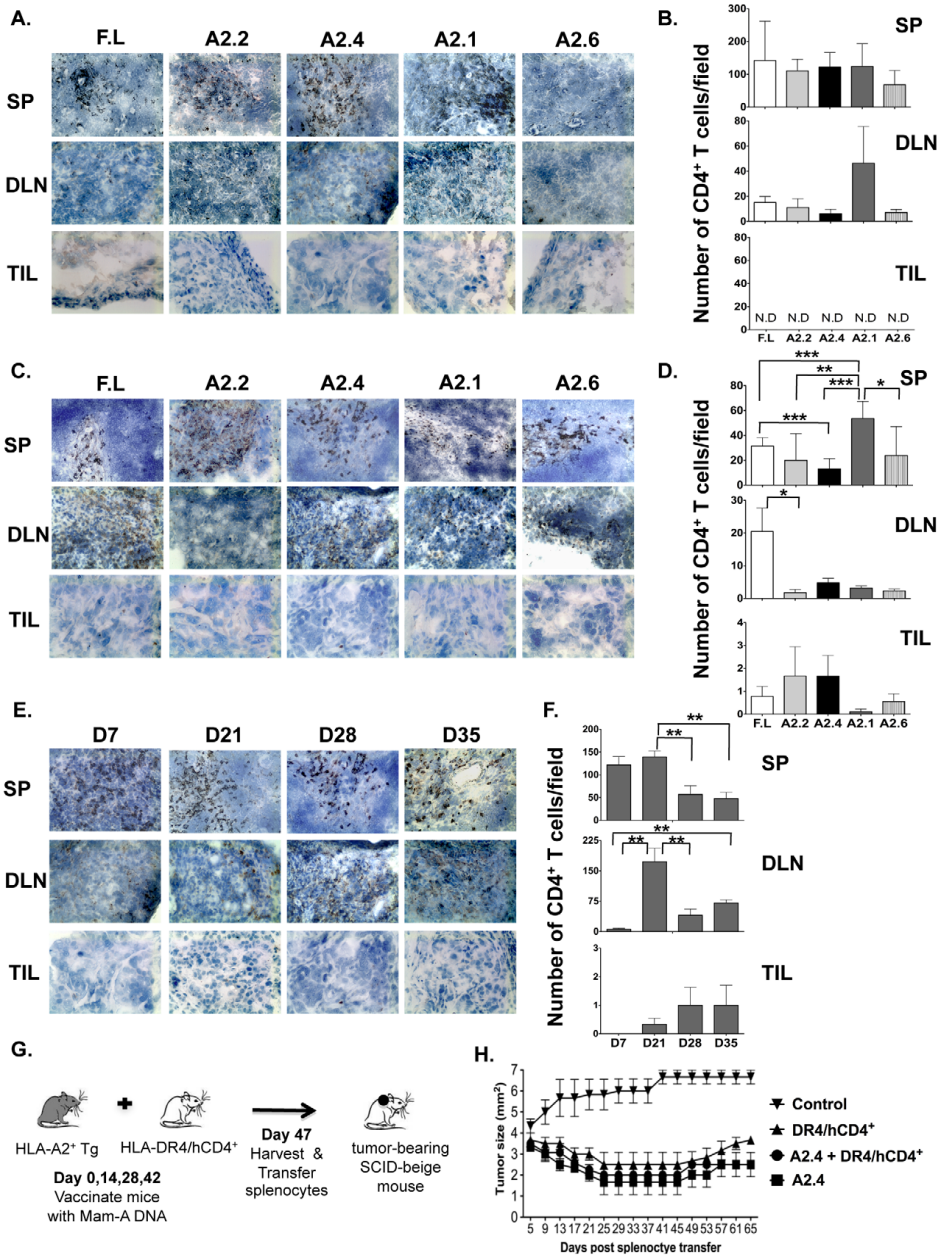
CD4 T cells fail to infiltrate the tumor by day 7. SP, DLN and TIL sections from SCID-beige mice receiving splenocytes from HLA-A2^+^ transgenic mice vaccinated with cDNA encoding either full-length or Mam-A epitopes were analyzed by immunohistochemistry for the presence of CD4 T cells (A) 7 days and (C) 28 days post transfer (magnification of 20×). Morphometric analysis was also performed (B) 7 and (D) 28 days post transfer and results are expressed as the mean (±SEM) of 3 different experiments. ND = none detected (E–F) SP, DLN and TIL tissue sections from tumor inoculated SCID-beige mice that received splenocytes from HLA-A2^+^ transgenic mice vaccinated with cDNA encoding Mam-A2.4 were analyzed for the presence of CD4 T cells on days 7, 21, 28 and 35 post spleen cell transfer. (G–H) HLA-DR4^+^/hCD4^+^ transgenic mice and HLA-A2 transgenic mice were vaccinated i.m a total of 4 times, separated by 2 week intervals, with 100 µg Mam-A full-length or Mam-A2.4 specific cDNA (respectively). Five days after the last vaccination spleen cells were harvested from full-length cDNA vaccinated HLA-DR4^+^/hCD4^+^ transgenic mice and Mam-A2.4 cDNA vaccinated HLA-A2^+^ transgenic mice. 1×10^7^ spleen cells from either Mam-A2.4 vaccinated mice, HLA-DR4^+^/hCD4^+^ vaccinated mice or 1×10^7^ spleen cells from both Mam-A2.4 and HLA-DR4^+^/hCD4^+^ vaccinated were injected i.p into SCID-beige mice bearing human breast tumors derived from the AU-565 breast cancer cell line. Tumor-bearing SCID-beige mice without spleen cell transfer were used as controls. Tumor regression was monitored using calipers every four days (n = 4 mice/group). Data are representative of 2 experiments. ^*^ P<0.05, ** P <0.01 and *** P<0.0001.

As CD4 T cells are often shown to be essential for tumor rejection [25], [26], [27], [28], we wanted to determine whether CD4 T cells control the recruitment of CD8 T cells indirectly through modifying the local tumor environment. Similar to CD8 T cells, immunohistochemical analysis showed that CD4 T cells infiltrated the spleen and DLN but in contrast to CD8 T cells there were no CD4 T cells detected in the tumor by day 7 (Fig. 5a–5b). However by day 28 post adoptive transfer, CD4 T cells had infiltrated the tumors (Fig. 5c–5d). Additionally, we analyzed tumors from mice that received spleen cells from Mam-A2.4 DNA vaccinated mice and found that CD4 T cells had infiltrated the tumor by day 21 and persisted at least until day 35 post splenocyte transfer (Fig. 5e–5f). Morphometric analysis for CD4 T cells further confirmed these findings (Fig. 5f). These data reveal that early infiltration of CD4 T cells is not required for the initial recruitment of Mam-A specific CD8 T cells into the tumor. To further investigate the role of CD4 T cells in our model we wanted to determine if increasing the number of tumor-specific CD4 T cells would enhance tumor regression and or maintain the tumors in check for longer. Therefore, we utilized HLA-DR4^+^/hCD4^+^ mice, which recognize MHC class II antigens in the context of human HLA-DR4 and co-express human CD4. These HLA-DR4^+^/hCD4^+^ mice were vaccinated four times with DNA encoding for full-length Mam-A, which also encodes for the HLA-DR4 peptides. We then transferred spleen cells obtained from HLA-DR4^+^/hCD4^+^ mice (which contained ∼2×10^4^ Mam-A specific CD4 T cells, determined by intracellular IFN-γ staining, Fig S4) along with spleen cells from HLA-A2^+^ transgenic mice vaccinated with Mam-A2.4 DNA into SCID-beige tumor bearing mice and examined tumor growth (Fig. 5g). The transfer of Mam-A specific CD4 T cells together with Mam-A specific CD8 T cells did not enhance tumor regression when compared to spleen cells from Mam-A2.4 DNA vaccinated mice alone (Fig. 5h). In addition enhanced tumor regression was not observed when CD4 T cells were depleted prior to adoptive transfer of spleen cells from Mam-A2.4 DNA vaccinated mice (Fig S3). Taken together these data indicate that while CD4 T cell help is not required for the recruitment of Mam-A specific CD8 T cells to the tumor site and additional help does not enhance the functional ability of the Mam-A specific CD8 T cells.

### CD8 T cells from mice vaccinated with Mam-A2.4 DNA in combination with a single dose of total body irradiation leads to long-term tumor regression and prevents tumor re-growth

**Figure 6:**
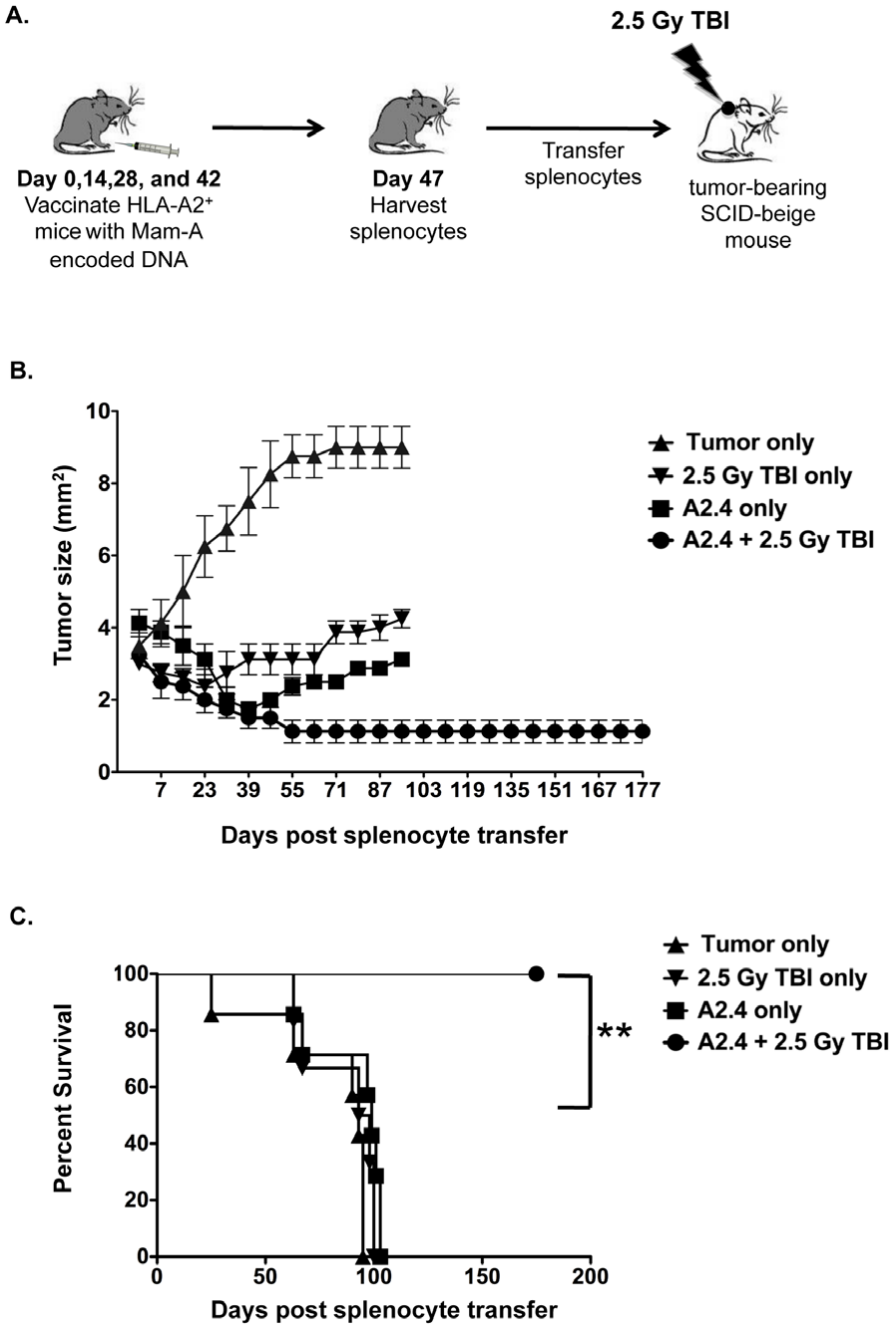
A single low dose of total body irradiation in combination with adoptive transfer of Mam-A2.4 CD8 T cells eliminates tumor and prevents relapse. (A) HLA-A2^+^ transgenic mice were vaccinated on days 0, 14, 28 and 42, with 100 µg Mam-A2.4 cDNA. Five days after the last vaccination, splenocytes were harvested and transferred i.p into tumor bearing SCID-beige mice immediately following the administration of 2.5 Gy TBI. (B) Tumor regression was monitored in SCID-beige mice that received either tumor only, 1×10^7^ Mam-A2.4 specific spleen cells alone, TBI (2.5 Gy) only or 1×10^7^ Mam-A2.4 specific spleen cells and TBI. Tumor regression was monitored using calipers every four days (n = 4 mice/group). (C) Survival curve of tumor-bearing mice. Day 0 indicates day of splenocyte transfer and TBI treatment. Data are representative of 3 independent experiments. **P<0.01.

Studies have shown that TBI immediately before the transfer of splenocytes is known to reduce suppressive cells found within the tumor microenvironment as well as remove cytokine sinks and cause an increase in danger signals [11], [12], [13]. We therefore assessed whether combining a single low dose of TBI with Mam-A2.4 DNA vaccination could induce long-term tumor regression and prevent relapse. SCID-beige mice bearing large established tumors were treated with a single low dose of TBI (2.5 Gy) immediately prior to receiving splenocytes from Mam-A2.4 vaccinated HLA-A2^+^ transgenic mice. Tumor regression was compared to that seen in tumor-bearing SCID-beige who received Mam-A2.4-specific splenocytes without TBI (Fig. 6a). TBI in combination with adoptive transfer of Mam-A2.4 splenocytes not only enhanced the effects of DNA vaccination, evident by the continual regression seen up until day 67 after transfer, but more importantly, prevented tumor re-growth up to day 175 post splenic transfer at which time the animals were sacrificed (Fig. 6b–c). The effects of TBI alone in tumor-bearing mice was also analyzed and while initial tumor regression was observed, the tumors quickly began growing back almost reaching their original size by day 95, by which time animals from all control groups were severely ill and sacrificed. This reiterates the need for combination therapy, as it confirms that in addition to TBI therapy you also need to adoptively transfer tumor-specific CD8 T cells to induce an effective anti-tumor immune response that prevents tumor escape.

### Total body irradiation reduces MSR1 and lipid uptake by tumor resident dendritic cells

**Figure 7:**
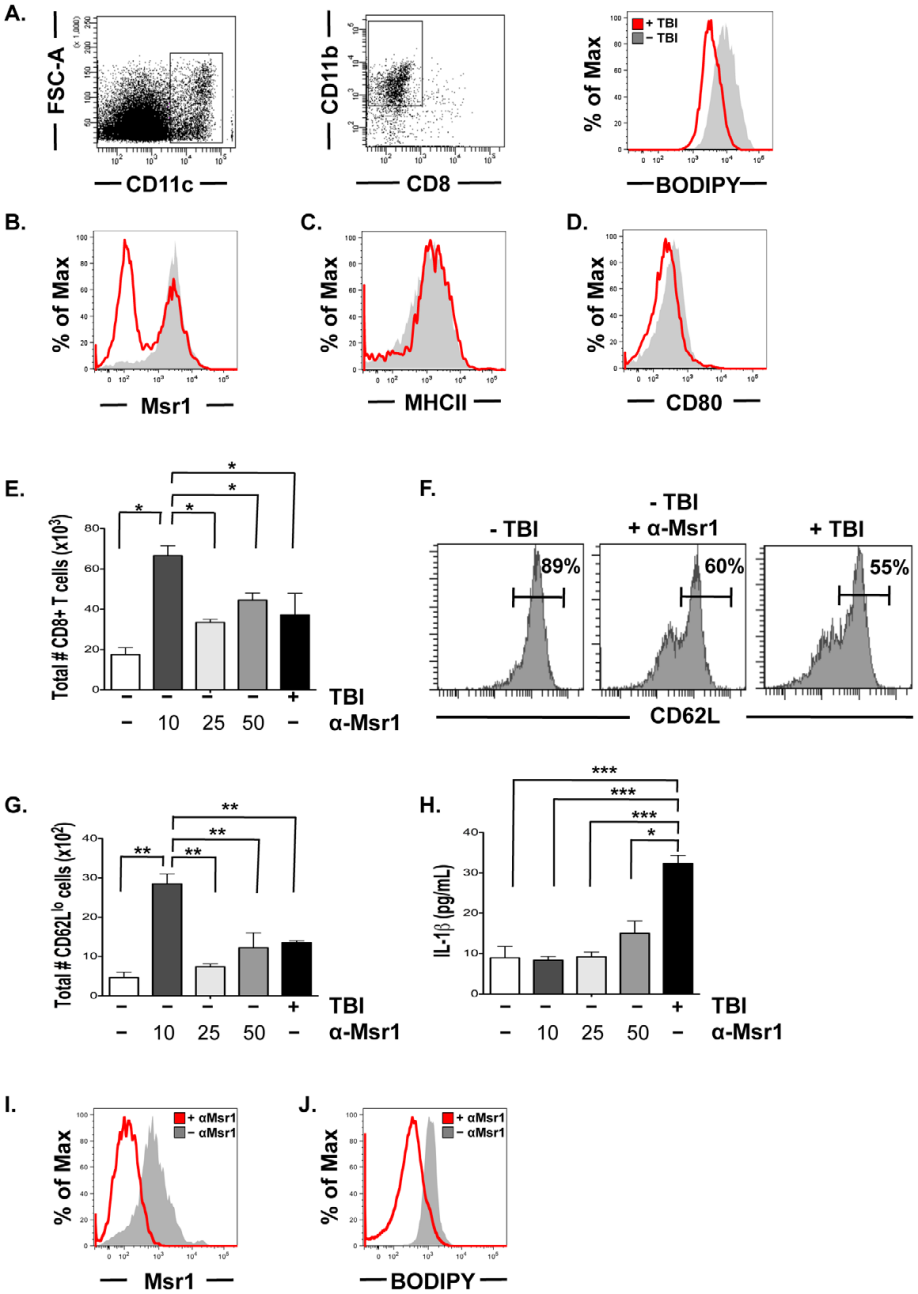
Irradiation reduces Msr1 and lipid uptake by tumor resident DCs. (A) Lipid levels were measured in CD11c+CD11b+ tumor resident DCs isolated from either non- irradiated tumor bearing SCID-beige mice or tumor bearing SCID-beige mice that received TBI (2.5 Gy) 1 day prior. (B) Expression of Msr1 in tumor resident DCs from either non-irradiated or irradiated mice. (C) Expression of MHC class II and (D) CD80 on tumor resident DCs from either irradiated or non-irradiated mice. (E–H) 1×10^3^ CD11c^+^CD11b^+^ tumor resident DCs from non- irradiated tumor bearing SCID-beige mice were incubated with anti-Msr1 (10,25 or 50 µg/ml) and cultured with 2×10^4^ Mam-A2.4 specific CD8 T cells, tumor-explant supernatants, and stimulated with Mam-A specific peptides. Control cultures received CD11c+CD11b+ tumor resident DCs from irradiated tumor bearing SCID-beige mice. (E) The total number of CD8 T cells after 5 days of culture with tumor explants supernatants. (F) CD62L expression on CD44^hi^ CD8 T cells. (G) The total number of CD62L^lo^ CD8 T cells in the cultures with tumor explants supernatants. (H) Culture supernatants were harvested and the levels of mouse IL-1β were measured by ELISA (*** P<0.001, * P<0.05). (I–J) CD11c^+^CD11b^+^ tumor resident DCs from non-irradiated tumor bearing SCID-beige mice were cultured for 4 hours in tumor explants supernatants with or without the addition of 10 µg/ml anti-Msr1 and then analyzed for the expression of (I) Msr1 and (J) BODIPY. Each group includes 3–6 mice. Results are representative of 2–3 experiments. All experiments were done when tumors were approximately 4×4 mm^2^ in size (23–28 days post tumor-cell inoculation). ^*^ P<0.05, ** P<0.01 and *** P<0.0001.

In an effort to understand the mechanism by which TBI provides the necessary cues for the transferred Mam-A2.4 specific CD8 T cells to induce a productive anti-tumor response, we examined if low dose TBI has an effect on lipid uptake by resident tumor DCs using the lipophilic fluorescent dye BODIPY [29]. Analysis of lipid content from CD11c+CD11b+ DCs isolated from tumors of irradiated mice (2.5 Gy) showed a more than two-fold decrease in BODIPY staining on tumor resident DCs compared to DCs isolated from non irradiated tumor bearing mice (MFI 3196 and 8867, respectively)(Fig. 7a). In contrast, we did not see any down-regulation of BODIPY from DCs isolated from either the spleen or tumor draining lymph nodes of irradiated mice, compared to non- irradiated hosts (data not shown). We next evaluated the level of expression of the macrophage scavenger receptor (Msr1), which has been shown to be up regulated in tumor resident DCs and responsible for the increased lipid uptake by DCs in tumor bearing hosts [29]. DCs isolated from tumors of mice that received low dose TBI had twofold less protein expression of Msr1 than control DCs isolated from tumors of non-irradiated mice (Fig. 7b). Additionally, low dose TBI did not increase the expression of the major histocompatibility complex (MHC) class II (Fig. 7c) or the co-stimulatory molecule CD80 on tumor resident DCs (Fig. 7d) or DCs located in the spleen or draining lymph nodes.

We next wanted to determine if Msr1 impacts the activation and proliferation of Mam-A2.4 specific CD8 T cells. For these experiments DCs were isolated from established tumors and incubated with Mam-A2.4 splenocytes, Mam-A2.4 specific peptide and tumor-explant supernatants along with 0, 10, 25 or 50 µg/ml of anti-Msr1. Control cultures received DCs from tumors that had been treated with 2.5 Gy and incubated with Mam-A2.4 splenocytes without anti-Msr1. Five days later the total number of CD8 T cells in cultures treated with anti-Msr1 was significantly greater than the total number of CD8 T cells in cultures that did not receive Msr1 antibody and comparable to cultures that received DCs from irradiated mice (Fig. 7e). In addition, cultures that were incubated with 10 µg of anti-Msr1 induced more CD8 T cells than cultures that received either 25 or 50 µg/ml of anti-Msr1 (Fig. 7e). Analysis of CD62L expression revealed that more CD8 T cells had been activated, evidence by the down-regulation of CD62L, in the cultures that received non-irradiated DCs and anti-Msr1 compared to control wells that received no anti-Msr1 (11% and 40%, respectively) and comparable to cultures that received irradiated DCs (45%) (Fig. 7f). Interestingly, the total number of CD62L^lo^ CD8 T cells was greater in cultures that received 10 µg/ml of Msr1 with non-irradiated DCs compared to cultures that received non-irradiated DCs (Fig. 7g). In contrast, the levels of IL-1β were significantly higher in the cultures that received the irradiated DCs compared to those cultures that received non-irradiated DCs and anti-Msr1 (Fig. 7h). To determine if blocking Msr1 directly affects lipid uptake we incubated tumor-derived DCs with tumor explants supernatants and anti-Msr1 for 4 hrs. This resulted in the down-regulation of Msr1 (Fig. 7i) and at least a four-fold decrease in BODIPY staining on the tumor-derived DCs that were incubated with anti-Msr1 compared to those tumor-derived DCs that did not receive the Msr1 antibody (MFI 2669 and 630, respectively)(Fig. 7j).

## Discussion

While effector CD8 T cells are capable of exerting anti-tumor responses they often fail to control tumor growth long-term. Early adoptive transfer studies using effector T cells that were activated *in vitro* with either anti-CD3 mAb or tumor antigen and then expanded with large quantities of IL-2 [30], [31] were found to have a terminal effector phenotype, characterized by a loss of CD62L and the expression of the senescence marker, KLRG1, as well as a decrease in proliferative capacity and IL-2 production [32], [33] . Hence these T cells were found to have short-term anti-tumor capabilities. Based on these findings, it was then proposed that memory CD8 T cells could be a better alternative for improving adoptive immunotherapy due to their ability to survive, proliferate and traffic to the tumor site and respond more effectively. Memory T cells are often divided into two populations, known as T_CM_ and T_EM_, although it remains controversial as to which memory CD8 T cell population provides the best anti-tumor immune response [32], [34], [35], [36]. One study reported that the in vitro generation of T_CM_ generated in the presence of IL-15 was far more effective at eradicating large, established tumors [34]. While others have reported that the presence of T_EM_ in adoptively transferred CD8 T cells correlates with an anti-tumor immune response [32], [35], [36]. T_CM_ are known to expand well in response to secondary activation and to survive long-term. Based on these qualities, T_CM_ could be beneficial for preventing relapse and eradicating large tumors. T_EM_ cells on the other hand preferentially locate to non-lymphoid tissue act as sentinel responders and have immediate cytotoxic activity [37]. This T_EM_ population is more likely to act rapidly to halt the growth of existing tumors in peripheral tissue. More importantly, the location of the tumor is most likely going to dictate which memory CD8 T cell population will provide the most successful anti-tumor response.

Our results demonstrate that Mam-A specific CD8 T cells generated by DNA vaccination *in vivo* result in a heterogeneous CD8 T cell population consisting of both T_CM_ and T_EM_ like cells. Furthermore, upon transfer into tumor bearing mice Mam-A2 specific CD8 T cells which accumulate and survive longer predominantly become more like an effector memory T cell expressing low levels of both CD62L and CD27 while maintaining high levels of CD127. We also found that these Mam-A2 (Mam-A2.4) specific CD8 T cells secrete significantly more IFN-γ and TNF-α when stimulated *ex vivo* on both 7 and 28 days post transfer when compared to CD8 T cells specific for the other Mam-A epitopes or full length Mam-A. Furthermore, Mam-A2.4 specific CD8 T cells express more granzyme B, localized to the tumor site in higher frequencies than CD8 T cells specific for the other Mam-A epitopes and resembled memory cell in that they expressed high levels of CD127 and CD122. Nonetheless, even with all these anti-tumor attributes the adoptive transfer of these Mam-A2.4 specific cells was not enough to prevent tumor recurrence.

Many tumor models also require help from CD4 T cells to achieve full tumor rejection [26], [28] [38]. Our model showed a lack of CD4 T cell infiltration into the tumor at both early and late time points indicating the Mam-A specific CD8 T cells do not require further help to enter the tumor microenvironment and cause tumor regression. Additionally, enhanced tumor regression was not observed when we co-transferred splenocytes from Mam-A vaccinated DR4/hCD4 mice and Mam-A specific CD8 T cells from HLA-A2 transgenic mice. Together these data reveal that in our model, where CD4 T cells are present at the time of priming, CD8 T cells do not require additional signals from CD4 T cells to gain access to the tumor.

While we show that the Mam-A2.4 specific CD8 T cells secreted more IFN-γ, TNF-α and Granzyme B compared to either the full-length or the other epitope Mam-A specific CD8 T cells, this adoptive T cell therapy failed to prevent tumor relapse. While considerable effort has gone into developing cancer vaccines that induce a high frequency of memory CD8 T cells, little emphasis has been devoted to improving the functional qualities of the anti-tumor memory T cells. Thus to improve the functional attributes of the Mam-A2.4 CD8 T cells we treated SCID-beige mice bearing large established tumors with a single low dose of TBI [12] immediately prior to adoptive transfer of Mam-A specific CD8 T cells. The use of TBI is known to impact tumor growth and because of the undesirable side effects observed with higher doses or multiple low doses we chose to use a single low dose of TBI which when combined with adoptive transfer resulted in the long-term control of tumor growth and prevented tumor recurrence. The mechanism responsible for the effect of TBI on adoptively transferred CD8 T cells has been shown to be via the increase in danger signals combined with the removal of cytokine sinks, as well as the removal of suppressive cells [11], [12]. Our tumor model uses a recipient SCID-beige mouse that lacks B, T and NK cells. Therefore, as NK and T cells are the predominant cell types that require γC cytokines it is unlikely that TBI is removing cytokine sinks in our model. In addition, TBI could have direct effects on the tumor that would impact tumor growth. We do show that tumor growth is affected by this single low dose of TBI however these tumors eventually escape and continue to grow. Recently it was reported that DCs taken from tumor bearing mice or individuals were functionally defective due to an increase in lipid uptake by these DCs [29]. Our data show that TBI induced the down-regulation of Msr1 and prevents tumor resident DCs from lipid uptake thereby potentially increasing the functional capacity of the adoptively transferred Mam-A specific CD8 T cells to induce tumor regression and prevent relapse. Furthermore, we show that blocking Msr1 on tumor derived DCs in vitro induces the proliferation and expansion of Mam-A specific CD8 T cells in greater numbers than cultures that received irradiated tumor derived DCs. However, in our model we cannot rule out the possibility that TBI is also acting to increase toll-like receptor signaling and/or induce microbial translocation, as described in a melanoma model [11], [12]. Another potential mechanism by which the quality of priming of Mam-A2.4 specific CD8 T cells may be induced during the treatment of tumor derived DC with irradiation could be via the induction of IL-1β. However, the immunogenicity seen with the inhibition of Msr1 on tumor derived DCs is not through the induction of IL-1β, as we show that irradiated tumor derived DCs produce significantly more IL-1β than non-irradiated tumor derived DCs incubated with either no, 10,25 or 50 ug/ml of Msr1. Thus we propose an additional mechanism of action of TBI is through the down regulation of Msr1 which results in the inhibition of lipid uptake by tumor resident DCs thus enabling them to present tumor antigen more efficiently.

In conclusion, we show that adoptive transfer of Mam-A2 specific CD8 T cells generated by DNA vaccination with all epitopes induces regression of established tumors but fails to maintain this anti-tumor response long term. However, when Mam-A2 specific CD8 T cells are combined with a single low dose of TBI this treatment induced not only regression of established tumors but more importantly prevented tumor recurrence. While we demonstrate that Mam-A2.4 is the most efficacious epitope, being able to sustain the anti-tumor response the longest, we recognize that due to the limitations of our model system (i.e., the immunodominant epitope could be influenced by impaired coreceptor interactions due to murine CD8 not being able to interact normally with human HLA class I molecules) that this might not translate directly into the clinical setting. Nonetheless, this model does demonstrate that epitope vaccination does work in combination with TBI. The exact mechanism(s) by which TBI impacts tumor immunity is unclear. In our model we propose that the low dose of TBI used is acting through several mechanisms that may not be mutually exclusive. Firstly, a single low dose of TBI alone is able to induce regression of established tumors by impacting tumor growth directly. Secondly, this single low dose of TBI impacts the biology of tumor derived DCs by reducing both Msr1 expression and lipid uptake both of which have been shown to affect the ability of DCs to present tumor antigen. Thus when TBI is combined with the adoptive transfer of Mam-A specific CD8 T cells, TBI slows the growth of tumor cells directly and at the same time increases the ability of tumor derived DCs to present Mam-A specific tumor antigen to the adoptively transferred Mam-A specific CD8 T cells which results in the prevention of tumor recurrence. In summary, we propose that adoptive transfer of Mam-A2 CD8 T cells encompassing a heterogeneous memory phenotype including CD62L^hi^/CD27^hi^, CD62L^lo^/CD27^hi^ and CD62L^lo^/CD27^lo^ together with low dose TBI, which effects both the rate of tumor growth and the ability of tumor derived DCs to present tumor antigens to tumor specific CD8 T cells, could be used as a therapy to effectively treat breast cancer patients.

## Materials and Methods

### Mice

C57BL/6 mice carrying the HLA-A*02:01 gene (HLA-A2^+^) were obtained from Jackson Laboratories. Severe combined immunodeficient (SCID) beige mice were purchased from Charles River Laboratories. C57BL/6 mice carrying the HLA-DRB1*0401 and human CD4 genes (HLA-DR4^+^/hCD4^+^) were generously provided by Dr G. Sondersrup via Dr. Tibor Glant (Department of Orthopedic Surgery, Rush University Medical Center). All protocols followed the guidelines approved by the Rush Institutional Animal Studies Committee.

### Mam-A cDNA constructs

The full-length Mam-A cDNA was generously provided by Dr. Timothy P. Fleming (Department of Surgery, Washington University School of Medicine, St. Louis, MO). The Mam-A cDNA was cloned into the PCI-neo vector (Promega, Madison, WI). Four cDNA constructs containing the coding sequence for a Kozac site (CCAC) + a start codon (ATG) + the epitope cDNA sequences for the Mam-A2.1, Mam-A2.2, Mam-A2.4, or Mam-A2.6 epitopes + a stop codon (TAG) were cloned into the pcDNA3.1+ vector (Invitrogen, Carlsbad, CA) (See Table 1).

**Table 1:** cDNA constructs for the Mammaglobin-A epitopes.

Peptide	AA position	AA Sequence	AA number
**Mam-A2.1:**	83–92	M-LIYDSSLCDL	10
CACC-ATG-TTG ATC TAC GAC TCC AGC CTG TGC GAT CTC-TAG			
**Mam-A2.2:**	2–10	M-KLLMVLMLA	9
CACC-ATG-AAG TTG CTG ATG GTG CTG ATG CTC GCC-TAG			
**Mam-A2.4:**	66–74	M-FLNQTDETL	9
CACC-ATG-TTC CTC AAC CAG ACA GAC GAG ACC CTG-TAG			
**Mam-A2.6:**	81–89	MQLIYDSSL	9
CACC-ATG CAG CTG ATC TAC GAC TCC AGC CTC-TAG			
**Kozac Site:** CACC**; Start Codon:** ATG (not added in Mam-A2.6 as sequence begins with a Start Codon)**; Stop Codon:** TAG			

### Mam-A Peptides and Mam-A cDNA vaccination

Mam-A-derived peptides that bind the HLA-A2 molecule (Mam-A2.1-Mam-A2.7) or HLA-DR4 (Mam-DR4.1, DR4.2, DR4.3, DR4.6, DR4.7 and DR4.8) were purchased from Sigma Aldrich (St. Louis, MO). Full-Length Mam-A cDNA was purified using an Endotoxin free plasmid purifying kit (Qiagen, Valencia, CA). HLA-A2^+^, or HLA-DR4^+^/hCD4^+^ transgenic mice were injected intramuscularly with 100 µg of Mam-A-derived cDNA (epitope or full-length). Mice were vaccinated 4 times at 2-week intervals. 5 days after the last vaccination, spleens and lymph nodes were harvested for transfer into tumor-bearing SCID-beige mice. Expression of Mam-A cDNA in the vaccinated HLA-A2^+^ transgenic mice was analyzed by PCR after the 3^rd^ vaccination. The following primers were used: pcDNA3.1, 5′-GAGACCCAAGCTGGCTAGC-3′, MamA2.1, 5′-ATCGCACAGGCTGGAGTCG-3′, MamA2.2, 5′-CGAGCATCAGCACCATCA GC-3′, MamA2.4, 5′-GTCTCGTCTGTCTGGTTGAGG-3′, MamA2.6, 5′-TGGAGTCGTAGATCAG CTGC-3′.

### Breast Cancer cell lines

The human breast cancer cell line AU-565 (Mam-A^+^/HLA-A2^+^:01^+^) was purchased from the American Type Culture Collection (Manassas, VA) and cultured according to their instructions. The cell line was expanded and aliquots frozen down within the first week of receipt. Each experiment was performed using a fresh aliquot of AU-565 cells and checked for the expression of HLA-A2 by flow cytometry before use.

### Spleen cell preparations

Single cell suspensions were prepared from lymph nodes and spleens as previously described [10]. Lymphocytes were isolated from tumors by digestion with 150 U/ml collagenase (Worthington Biochemical, Lakewood, NJ) in RPMI containing 1 mM MgCl_2_, 1 mM CaCl_2_, and 5% fetal bovine serum (FBS), at 37°C for 30 min. Cells were washed and resuspended in staining buffer (PBS with 2% FBS and 0.01% NaN_3_).

### Tumor regression model

SCID-beige mice were inoculated subcutaneously (s.c.) with AU-565 breast cancer cells (1×10^7^) resuspended in 300 uL of BD Matrigel basement membrane (BD Biosciences, Franklin Lakes, MD). Once the tumors reached ∼4 mm^2^ in diameter, SCID-beige mice were reconstituted i.p. with 1×10^8^ spleen cells from either epitope or full-length Mam-A cDNA vaccinated mice. Tumor growth was measured with calipers every four days.

### Antibodies and flow cytometry

At the indicated times after tumor inoculation, lymphocytes were isolated and Mam-A-specific CD8 T cells were detected using tetramers to the Mam-A epitopes (A2.1–A2.6) provided by the NIH tetramer facility. For staining, lymphocytes were resuspended at a concentration of 1×10^6^–1×10^7^ cells/ml, incubation for 1 h with Mam-A –tetramers-APC plus the appropriate dilution of anti-CD122-FITC anti- CD27-PE, anti-CD8α-PerCPCy5.5, anti-CD11a, anti-CD62L-APC750 and anti-CD127-PB (all from eBioscience, San Diego, CA). Cells were washed, and fixed in 3% paraformaldehyde in PBS. Staining for MSR1 (Serotec) and BODIPY (molecular Probes) was done as described previously [11]. Relative fluorescence intensities were measured on a LSR II (BD Biosciences). Data were analyzed using FlowJo (Tree Star, Ashland, OR).

For intracellular staining, splenocytes were cultured for 5 hrs with 1 µg/ml GolgiPlug (BD Biosciences) and 40 µg/mL pooled Mam-A peptides (Mam-A2.1, A2.2, A2.4 and A2.6) (Sigma-Aldrich). Cells were stained for surface markers, then fixed and permeabilized using Cytofix/Cytoperm solution (BD Bioscience) for 20 min at 4°C. Cells were then stained, with anti-IFNγ-FITC anti-TNFα-APC anti-Granzyme B or the corresponding isotype controls (BD Biosciences). Finally, cells were washed twice and analyzed by Flow cytometry.

### Histological analysis

Tissues were fixed and frozen in OCT medium, cut into 10-mm sections, fixed for 10 minutes with cold ethanol and washed twice with PBS before being blocked for 5 minutes with 1% hydrogen peroxide. Sections were then blocked for 30 minutes with Avidin and Biotin (Vector Laboratories, Burlingame, CA) at room temperature prior to staining with primary antibodies for CD8 or CD4 (eBiosciences). Sections were then washed and incubated with biotin-conjugated F(ab')2 rat anti-goat IgG (Jackson ImmunoResearch Laboratories, West Grove, PA). The presence of CD8 and CD4 T cells within the tissues was detected with DAB (Sigma-Aldrich) and counter-stained with Gill's hematoxylin (Sigma-Aldrich).

### PCR analysis

RNA was either isolated from the AU-565 tumor cell line prior to transplant or from AU-565 tumors isolated 35 days after transplant sing the Qiagen RNeasy Mini kit (Qiagen, Valencia, CA). RT-PCR was performed on freshly isolated RNA using the high capacity RNA to cDNA kit (Applied Biosystems, Carlsbad, CA). 20 ug of cDNA was amplified using the Sigma RedExtract kit (Sigma-Aldrich) and the following primers were used: pcDNA3.1, 5′-GAGACCCAAGCTGGCTAGC-3 and Mam-A2.4, 5′-GTCTCGTCTGTCTGGTTGAGG-3′.

### In Vitro Msr1 blocking assay

Tumors from irradiated (2.5 Gy) or non-irradiated SCID-beige mice were harvested and CD11c^+^ DCs were purified by positive selection (Miltenyi, CA) (>85% CD11c^+^ by flow cytometry). CD8^+^ T cells from the spleens of Mam-A2.4 DNA vaccinated mice were purified by positive selection (Miltenyi, CA) (>80% CD8^+^ by flow cytometry) and then labeled with 5 mM CFSE at a concentration of 1 µL CFSE per 1×10^7^ cells/mL. The resulting CFSE-labeled CD8+ T cells were then cultured (1×10^5^/well) in a 96-well tissue plate (Becton Dickinson NJ) with tumor-explant supernatants in supplemented RPMI with CD11c+ DCs (1×10^4^ cells/well) and stimulated with Mam-A2.4 peptide (Invitrogen, CA 40 µg/mL). Msr1 blocking antibody was added to the corresponding wells at a concentration of 10, 25 or 50 µg/mL (Serotec NC). Cells were incubated at 37°C with 5% CO_2_ for 5 days before being harvested and analyzed via flow cytometry.

### Statistical analysis

Statistical significance was determined by Wilcoxon nonparametric tests. Differences in graft survival were assessed by means of 2-way log-rank (Mantel-Haenszel) test. Analyses were performed using the Prism 5.0 software (GraphPad, San Diego, CA) with significance set *a-priori* at *P*<0.05.

## Supporting Information

Figure S1 **Vaccination with Mam-A2.2 and Mam-A2.4 epitope DNA induces a heterogeneous population of CD8 T cells.** HLA-A2^+^ transgenic mice were vaccinated four times with full-length Mam-A or Mam-A2.1, Mam-A2.2, Mam-A2.4 or Mam- A2.6 epitope cDNA. (A) The expression of CD122 and CD127 on splenic Mam-A tetramer positive CD8 T cells was determined five days after the last vaccination. (B) CD8 T cells were analyzed for IFN-γ production after stimulation with 40 µg/ml of the corresponding Mam-A peptides. The percentage of antigen specific CD8 T cells that secrete IFN-γ in response to peptide stimulation is shown. (C) Expression of Foxp3 on CD25+ CD4 T cells five days after the last vaccination is shown. Data are representative of 3 experiments with at least 2–3 mice per experiment.
(TIF)

Figure S2 **HLA-A2^+^ transgenic mice were vaccinated i.m a total of 4 times.** Five days after the last vaccination 1×10^7^ Mam-A full-length or epitope specific spleen cells were harvested and injected i.p into tumor-bearing SCID-beige mice. Tumor regression was monitored in mice that received either splenocytes from unvaccinated mice, tumor alone, full-length Mam-A, Mam-A2.1 (left panel) or Mam-A2.2, Mam-A2.4 or Mam-A2.6 specific spleen cells (right panel). Tumor size was normalized and expressed as the percentage change from the initial tumor size. Results representative of 4 independent experiments with n = 4 mice/group.
(TIF)

Figure S3 **Depleting splenocytes of CD4 T cells prior to adoptive transfer does not enhance tumor regression.** HLA-A2 transgenic mice were vaccinated i.m a total of 4 times, separated by 2-week intervals, with 100 µg Mam-A2.4 encoded cDNA. Five days after the last vaccination spleens were harvested and 1×10^7^ whole splenocytes or splenocytes that had been CD4 T cell depleted by MACs column were injected i.p. into tumor bearing SCID-beige. Tumor regression was monitored in mice that received either: tumor alone, CD4 depleted Mam-A2.4 splenocytes, or Mam-A2.4 whole splenocytes (n = 4 mice/group).
(TIF)

Figure S4 **Mam-A specific CD4 T cells prior to adoptive transfer.** HLA-DR4 transgenic mice were vaccinated four times with full-length Mam-A and splenocytes removed and stimulated with a pool of 6 Mam-A DR4 peptides (10 µg/ml of each). A representative plot with the percent of IFN-γ producing CD44^hi^ CD4 T cells is shown.
(TIF)
