# Supplementary figures and images for: Adoptive Transfer of Mammaglobin-A Epitope Specific CD8 T Cells Combined with a Single Low Dose of Total Body Irradiation Eradicates Breast Tumors
Source: PLoS One. 2012 Jul 20;7(7):e41240. doi: 10.1371/journal.pone.0041240 (PMC3401129; doi:10.1371/journal.pone.0041240)

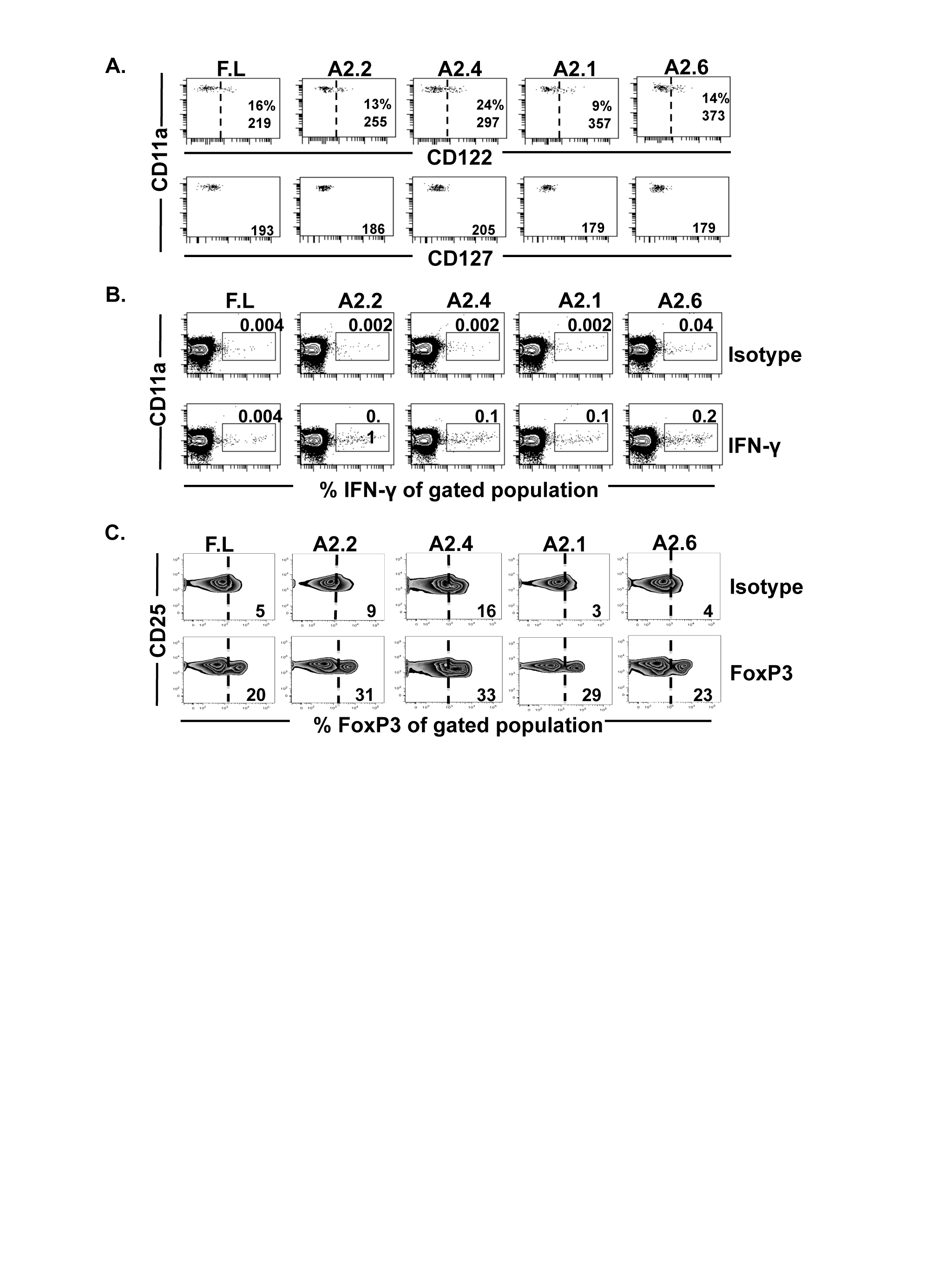

Supplement: Figure S1 — Vaccination with Mam-A2.2 and Mam-A2.4 epitope DNA induces a heterogeneous population of CD8 T cells. HLA-A2+ transgenic mice were vaccinated four times with full-length Mam-A or Mam-A2.1, Mam-A2.2, Mam-A2.4 or Mam- A2.6 epitope cDNA. (A) The expression of CD122 and CD127 on splenic Mam-A tetramer positive CD8 T cells was determined five days after the last vaccination. (B) CD8 T cells were analyzed for IFN-γ production after stimulation with 40 µg/ml of the corresponding Mam-A peptides. The percentage of antigen specific CD8 T cells that secrete IFN-γ in response to peptide stimulation is shown. (C) Expression of Foxp3 on CD25+ CD4 T cells five days after the last vaccination is shown. Data are representative of 3 experiments with at least 2–3 mice per experiment. (TIF) [file pone.0041240.s001.tif]

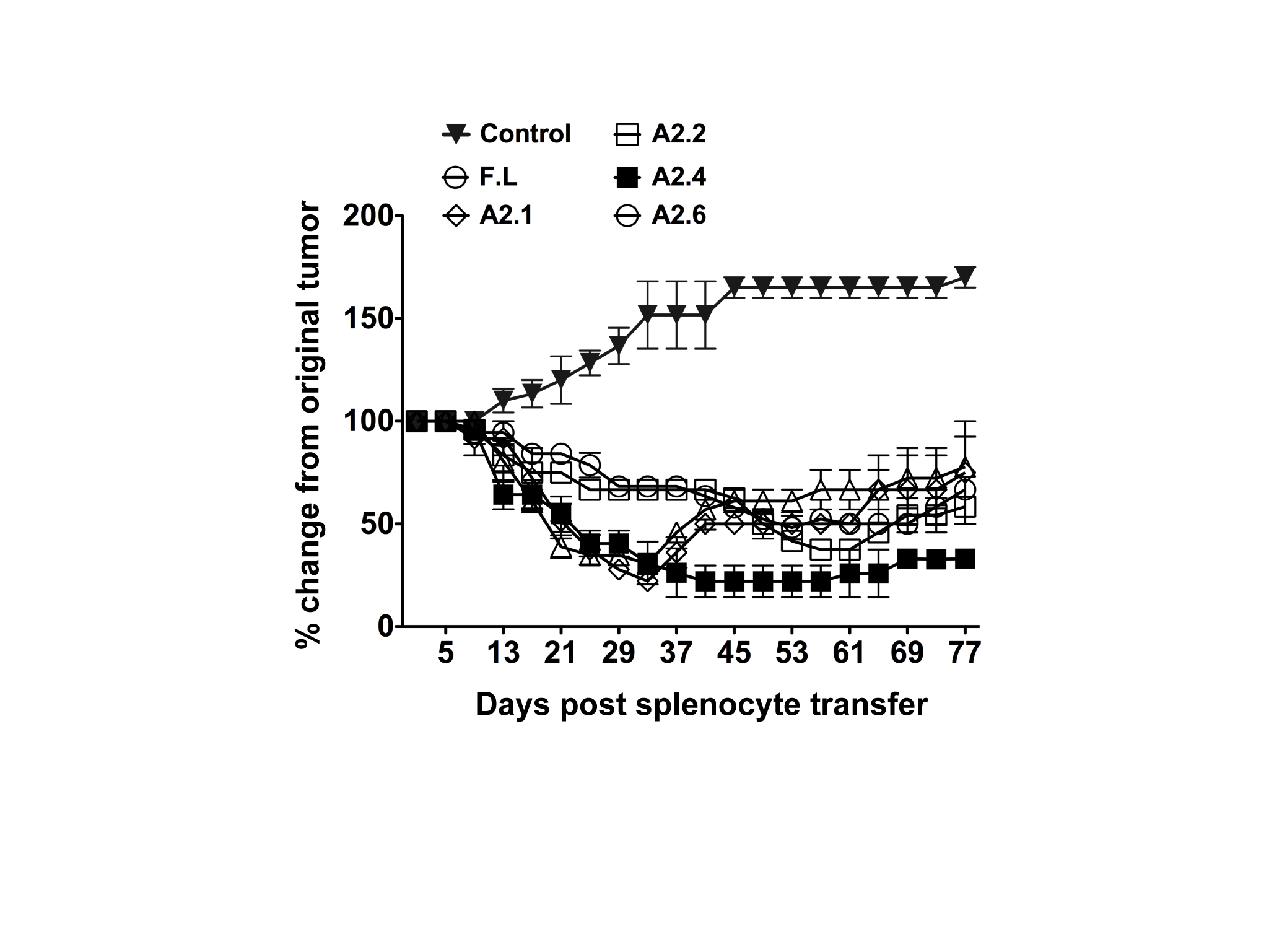

Supplement: Figure S2 — HLA-A2+ transgenic mice were vaccinated i.m a total of 4 times. Five days after the last vaccination 1×10^7^ Mam-A full-length or epitope specific spleen cells were harvested and injected i.p into tumor-bearing SCID-beige mice. Tumor regression was monitored in mice that received either splenocytes from unvaccinated mice, tumor alone, full-length Mam-A, Mam-A2.1 (left panel) or Mam-A2.2, Mam-A2.4 or Mam-A2.6 specific spleen cells (right panel). Tumor size was normalized and expressed as the percentage change from the initial tumor size. Results representative of 4 independent experiments with n = 4 mice/group. (TIF) [file pone.0041240.s002.tif]

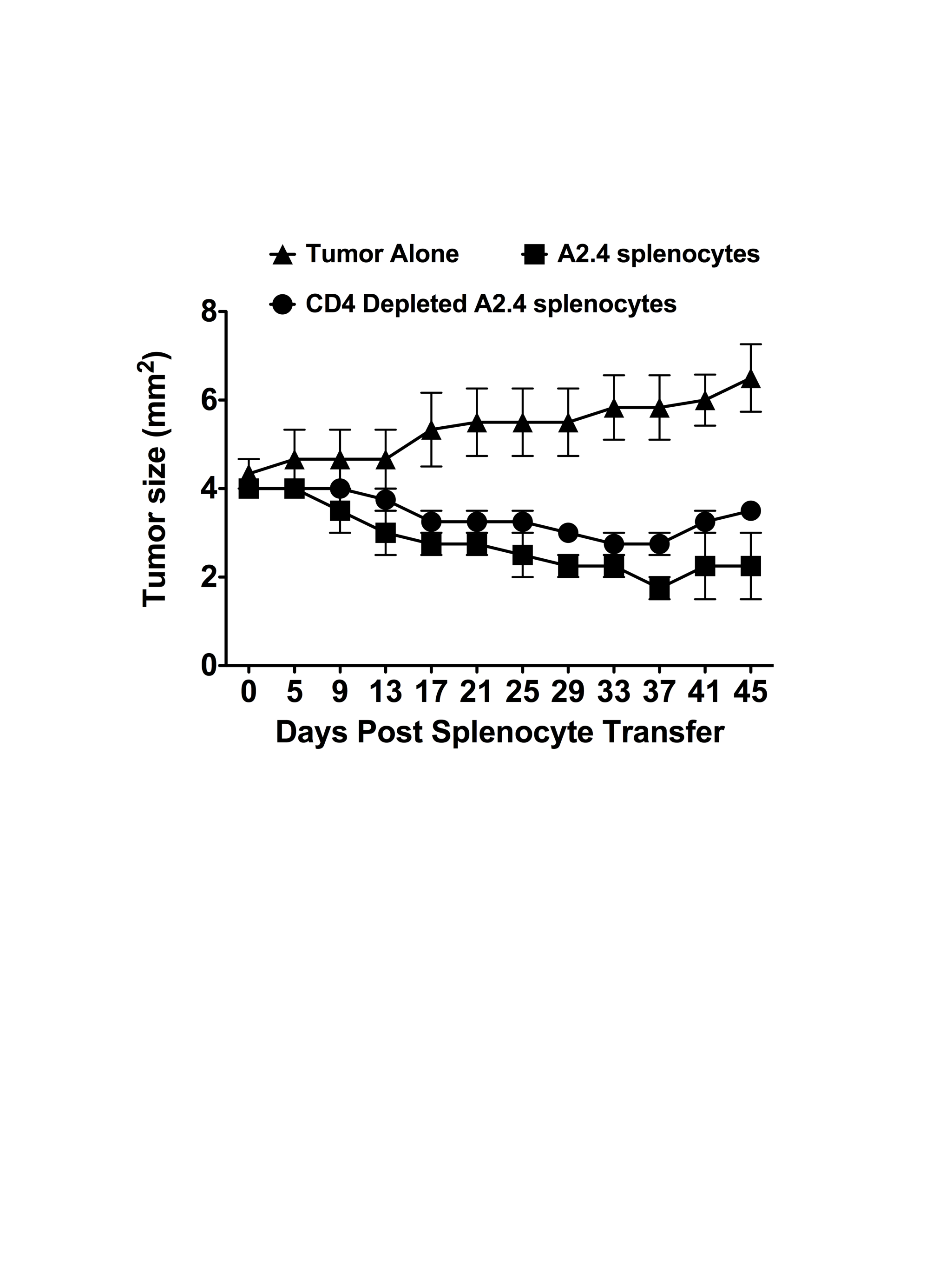

Supplement: Figure S3 — Depleting splenocytes of CD4 T cells prior to adoptive transfer does not enhance tumor regression. HLA-A2 transgenic mice were vaccinated i.m a total of 4 times, separated by 2-week intervals, with 100 µg Mam-A2.4 encoded cDNA. Five days after the last vaccination spleens were harvested and 1×10^7^ whole splenocytes or splenocytes that had been CD4 T cell depleted by MACs column were injected i.p. into tumor bearing SCID-beige. Tumor regression was monitored in mice that received either: tumor alone, CD4 depleted Mam-A2.4 splenocytes, or Mam-A2.4 whole splenocytes (n = 4 mice/group). (TIF) [file pone.0041240.s003.tif]

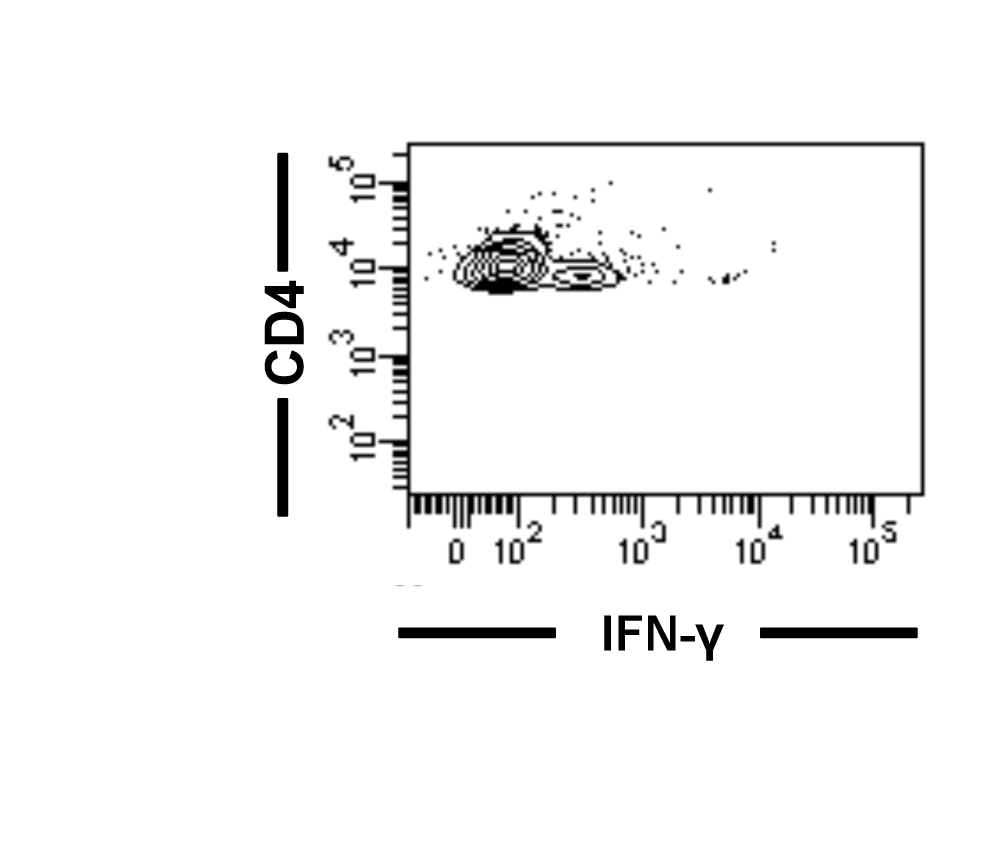

Supplement: Figure S4 — Mam-A specific CD4 T cells prior to adoptive transfer. HLA-DR4 transgenic mice were vaccinated four times with full-length Mam-A and splenocytes removed and stimulated with a pool of 6 Mam-A DR4 peptides (10 µg/ml of each). A representative plot with the percent of IFN-γ producing CD44hi CD4 T cells is shown. (TIF) [file pone.0041240.s004.tif]
